# Probabilistic ecological risk assessment of heavy metals in western Laizhou Bay, Shandong Province, China

**DOI:** 10.1371/journal.pone.0213011

**Published:** 2019-03-14

**Authors:** Xia Li, Wanqing Chi, Hua Tian, Yongqiang Zhang, Zichen Zhu

**Affiliations:** 1 The First Institute of Oceanography, State Oceanic Administration of China, Qingdao, China; 2 College of Marine Geosciences, Ocean University of China, Qingdao, China; 3 College of Marine Life Sciences, Ocean University of China, Qingdao, China; Duke University Marine Laboratory, UNITED STATES

## Abstract

Considering the serious land-based pollution and the weak water exchange ability of western Laizhou Bay, it is essential to conduct an ecological risk assessment of the pollutants in this area. In this study, the ecological risk caused by heavy metals deposited in the surface sediments and those resuspended in the seawater of western Laizhou Bay was evaluated using probabilistic approaches. First, the concentrations of seven heavy metals, namely As, Cd, Cr, Cu, Hg, Pb, and Zn, in the surface sediments and seawater of western Laizhou Bay were detected during the spring and autumn of 2016. The concentrations of As, Cd, Cr, Cu, and Pb were found to be at levels comparable to those in the other global coastal systems, while those of Hg and Zn were lower than those in other coastal areas. Next, an ecological risk assessment of heavy metals in the surface sediments was performed using a typical potential ecological risk index and refined by using a Monte Carlo simulation. The results suggested low risk for the heavy metals detected in the sediments of western Laizhou Bay, with the exception of Hg in September 2016, which showed a probability (0.03%) of moderate risk. Meanwhile, the aquatic ecological risk assessment of the heavy metals was performed by applying a combination of hazard quotient (HQ) and joint probability curve. While the ecological risk of Cd, Hg, and Pb was found to be acceptable, the HQs for Cr, Cu, and Zn were greater than 1, and the overall risk probability of their adverse effects was higher than 0.05, suggesting certain ecological risk. Specifically, in the case of As, the overall risk probability was lower than 0.05, suggesting that its ecological risk was acceptable, although its HQ was greater than 1. Thus, by applying the probabilistic approaches, the ecological risk of the heavy metals in western Laizhou Bay was better characterized in this study, avoiding both overestimation and underestimation of ecological risk.

## Introduction

Due to their poor biodegradability, easy bioaccumulation, and high toxicity, heavy metals discharged into the sea from different sources may pose serious threats to marine organisms. For example, the spore release of *Ulva pertusa* is inhibited by exposure to Cu, Cd, Pb, and Zn [1]; Cd arrests the molting of the estuarine crab *Chasmagnathus granulata* by preventing the normal peaking of the ecdysteroids needed for molting [2]; disorganization of epithelial cells is observed in the gills of Arctic charr (*Salvelinus alpinus*) after exposure to Hg (15 μg/L) for 12 h [3]; DNA damage is induced in marine bivalve mollusk (*Mytilus edulis*) by Cu exposure at a low concentration of 18 μg/L [4]; and the embryo development of *Ruditapes decussatus* is observed to be inhibited when the median effective concentration (EC_50_) values are 4.2 μg/L for Hg and 9.1 μg/L for Cu [5].

Laizhou Bay is the largest bay located in the Shandong Province of China. On the one hand, owing to the superior natural conditions, Laizhou Bay has become one of the most important centers of economic activities. Suitable temperature and salinity, as well as rich natural resources, make Laizhou Bay an important area for utilization and conservation of fishery resources. Besides, salt production, oil and gas exploitation, marine communications and transportation, and marine chemical engineering are rapidly developing in Laizhou Bay and its coastal areas. On the other hand, due to the specific natural conditions and the above-mentioned human activities, Laizhou Bay, especially the western region, has become one of the most polluted regions in China. As Laizhou Bay is a semi-closed sea, it exhibits a long period of water exchange. Thus, transport and diffusion of pollutants from the inner bay to the outer bay is very limited. Along the coast, more than ten rivers including the Yellow River, Xiaoqinghe River, Zhimaihe River, and Weihe River enter western Laizhou Bay, transporting high concentrations of heavy metals [6–10]; the Yellow River and Xiaoqing River alone carried 316 tons of heavy metals into Laizhou Bay in 2016 [11]. Hence, the need for an ecological risk assessment of the heavy metals in western Laizhou Bay is highlighted.

Previous studies evaluated the risk caused by heavy metal deposition in sediments of Laizhou Bay using typical risk assessment indices, including enrichment factor (EF), potential ecological risk index (PERI), and index of geo-accumulation (I_geo_) [12–16]. However, methods adopted by these studies are essentially single-point estimates where the risks might be either underestimated or overestimated due to the uncertainty of the risks. The United States Environmental Protection Agency (US EPA) has suggested the use of a Monte Carlo simulation to refine the ecological risk assessment. As a probabilistic approach, Monte Carlo simulation can produce a large quantity of random numbers conforming to a certain rule, which can be brought into a risk assessment model to quantitatively estimate the probabilities of specific levels of adverse biological effects [17], reflecting the uncertainty and variability in the risk assessment process [18, 19]. Furthermore, previous studies mainly focused on the risk posed by heavy metals only in the sediments of Laizhou Bay, while ignoring the water column. It is noteworthy that although the sediments are the main cause of heavy metal contamination in the marine environment, the heavy metals settled in the sediments may re-enter seawater through desorption and other mechanisms [20–23]. Therefore, an aquatic ecological risk assessment is also very important, which can be achieved by using an initial point estimate such as the hazard quotient (HQ, which is a comparison of the values of exposure concentrations and toxicant effects) [24–25] and higher level methods such as the joint probability curve (JPC) [17, 26].

In this paper, all the seven heavy metals (i.e., As, Cd, Cr, Cu, Hg, Pb, and Zn) included in both Marine Sediment Quality (GB 18668–2002) and Sea Water Quality Standard (GB 3097–1997) were detected in the surface sediments and seawater of western Laizhou Bay during the spring and autumn of 2016. Then, the ecological risk posed by these metals in the surface sediments was assessed by using a typical PERI followed by Monte Carlo simulation; and the aquatic ecological risk assessment of the heavy metals was achieved through a combination of the HQ and JPC.

## Materials and methods

### Sample collection and analysis

**Fig 1:**
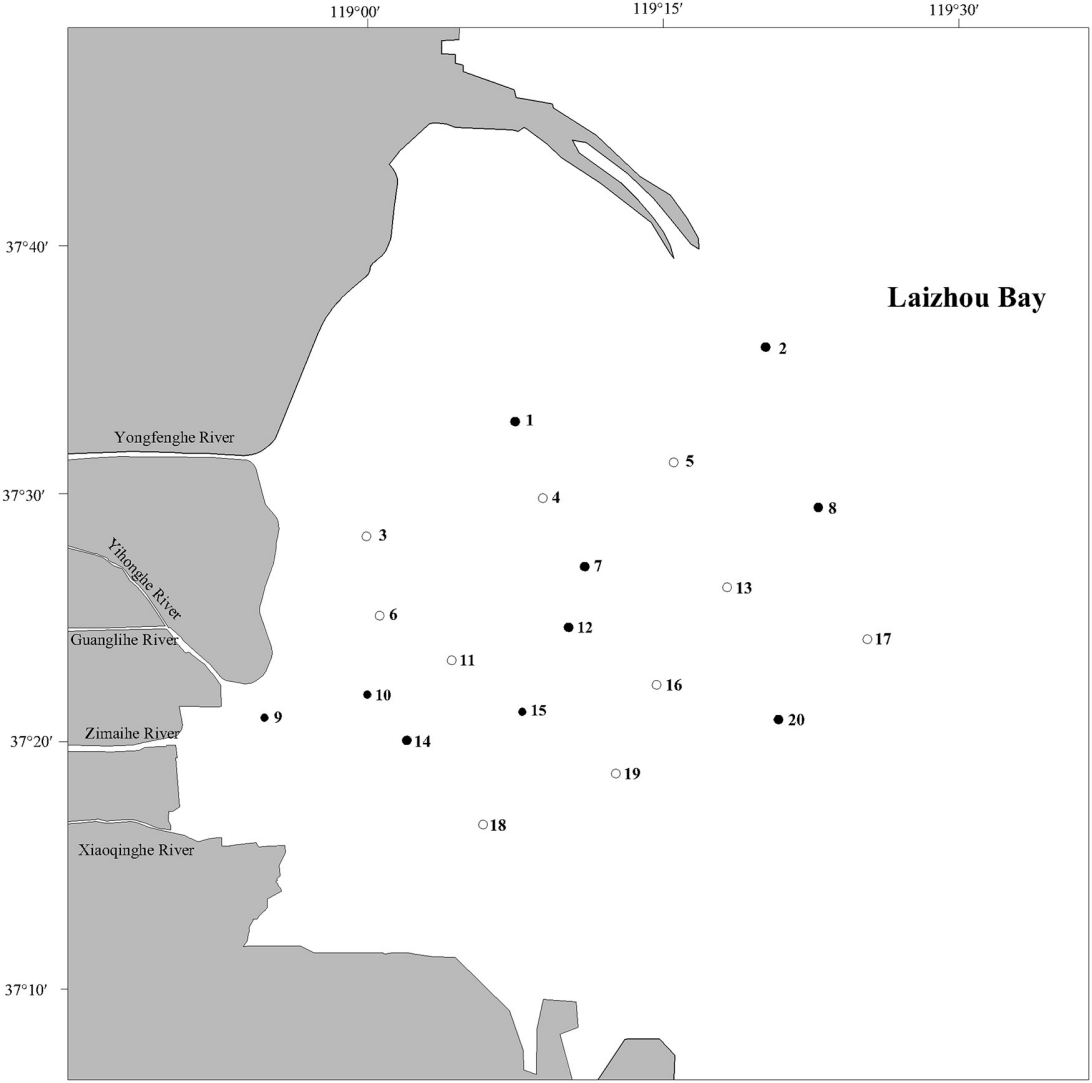
Map of sampling stations (● seawater and sediments, ○ seawater).

The field survey in this study was conducted based on the Marine Environmental Impact Assessment Project of Guangli Port Logistics Park, which was approved by Dongying Marine and Fisheries Bureau. Twenty sampling stations were set up in western Laizhou Bay (118°55′03.08″–119°25′28.78″ E, 37°16′50.22″–37°36′11.78″ N) in May (spring) and September (autumn) 2016 (Fig 1 and Table 1).

**Table 1:** Geographical information of sampling stations.

Sampling station	Geographical coordinate		Collected Sample
	East longitude	North latitude	
1	119°07′34.07″	37°33′06.75″	Seawater and sediments
2	119°20′13.70″	37°36′11.78″	Seawater and sediments
3	119°00′05.21″	37°28′25.78″	Seawater
4	119°09′00.39″	37°30′02.15″	Seawater
5	119°15′37.47″	37°31′30.81″	Seawater
6	119°00′48.36″	37°25′13.91″	Seawater
7	119°11′09.88″	37°27′17.26″	Seawater and sediments
8	119°22′57.71″	37°29′44.59″	Seawater and sediments
9	118°55′03.08″	37°21′03.78″	Seawater and sediments
10	119°00′13.83″	37°22′02.02″	Seawater and sediments
11	119°04′28.48″	37°23′27.69″	Seawater
12	119°10′22.40″	37°24′49.92″	Seawater and sediments
13	119°18′21.48″	37°26′29.29″	Seawater
14	119°02′14.68″	37°20′12.38″	Seawater and sediments
15	119°08′04.29″	37°21′24.34″	Seawater and sediments
16	119°14′50.00″	37°22′32.87″	Seawater
17	119°25′28.78″	37°24′25.94″	Seawater
18	119°06′07.75″	37°16′50.22″	Seawater
19	119°12′49.15″	37°18′57.00″	Seawater
20	119°21′01.18″	37°21′10.63″	Seawater and sediments

Surface sediment samples (0–5 cm sediment layer) were obtained by a grab sampler and collected using glass jars for Hg analysis and polythene bags for As, Cd, Cr, Cu, Pb, and Zn detection. Surface seawater samples (at a depth of 0.5 m) were collected using glass bottles (for Hg detection) and plastic bottles (for analysis of the other heavy metals). Three seawater samples and three sediment samples were collected from each station. The sealed sediment and water samples were sent to our laboratory for treatment and analysis, in accordance with the Specifications for Marine Monitoring (GB 17387.4–2007 and GB 17387.5–2007). The analytic techniques and the detection limits are shown in Table 2.

**Table 2:** Analytic techniques and detection limits.

Matter	Analytic technique	Detection limit	
		Seawater (μg/L)	Sediments (μg/kg)
As	Atomic fluorescence spectroscopy	0.5	0.06
Cd	Flameless atomic absorption spectroscopy	0.01	0.04
Cr	Flameless atomic absorption spectroscopy	0.4	2.0
Cu	Flameless atomic absorption spectroscopy	0.2	0.5
Hg	Atomic fluorescence spectroscopy	0.007	0.002
Pb	Flameless atomic absorption spectroscopy	0.03	1.0
Zn	Flame atomic absorption spectroscopy	3.1	6.0

### Toxicity data collection

Chronic toxicity data for the seven heavy metals with respect to their impact towards marine species were collected from the ECOTOX database (http://cfpub.epa.gov/ecotox/) and screened according to the criteria of reliability, relevance, and adequacy [27]. No observed effect concentration (NOEC) was adopted as the primary endpoint representing chronic toxicity, with maximum acceptable toxicant concentration (MATC) and lowest observed effect concentration/level (LOEC/LOEL) serving as a supplement. Data were adopted only from exposure experiment with adequate duration. To be specific, the exposure period should be ≥1 d for algae and invertebrates and ≥4 d for crustaceans, fish, mollusks, and worms. Overall, 336 chronic toxicity values of heavy metals with respect to marine species were available (Table 3). The data on the toxicity values for each pollutant with respect to all six major functional groups of the marine ecosystem were involved, meeting the requirement by the US EPA of at least eight families in three classes of tested organisms.

**Table 3:** Data size of available toxicity data of heavy metals towards marine species.

Functional group	As	Cd	Cr	Cu	Hg	Pb	Zn
Algae	12	12	16	38	6	8	10
Crustaceans	5	2	10	27	2	6	18
Fish	4	9	3	9	1	1	4
Invertebrates	1	6	4	15	3	1	2
Mollusks	1	16	3	25	6	11	15
Worms	0	6	2	9	2	2	3
Total	23	51	38	123	20	29	52

### Ecological risk assessment approach

#### Potential ecological risk index

According to Hakanson [28], the potential ecological risk of a given substance in the sediments was calculated as follows:

$${\text{E}}_{r}^{i}=T_{r}^{i}\times C_{f}^{i}=T_{r}^{i}\times C_{0}^{i}/C_{r}^{i}$$

where $E_{r}^{i}$ is the potential ecological risk factor of substance ‘‘*i*”, $T_{r}^{i}$ is the toxic response factor of substance ‘‘*i*” (which is 10 for As, 30 for Cd, 2 for Cr, 5 for Cu and Pb, 40 for Hg, and 1 for Zn [28]), $C_{f}^{i}$ is the contamination factor of substance ‘‘*i*”, $C_{0}^{i}$ is the measured concentrations in the sediments of substance ‘‘*i*”, and $C_{r}^{i}$ is the background reference level for substance ‘‘*i*”. Grade I of the Marine Sediment Quality (GB 18668–2002) was adopted as $C_{r}^{i}$ in this study. The following grades were used for the $E_{r}^{i}$ value: (I) low risk: *$E_{r}^{i}<40$*; (II) moderate risk: $40\leq E_{r}^{i}<80$; (III) considerable risk: $80\leq E_{r}^{i}<160$; (IV) high risk: $160\leq E_{r}^{i}<320$; (V) very high risk: *$E_{r}^{i}\geq 320$*.

*RI* represents the ecological risk for the sediment. It was calculated as the sum of $E_{r}^{i}$ and categorized into the following four classes: (I) low risk: *RI* < 150; (II) moderate risk: 150 ≤ *RI* < 300; (III) considerable risk: 300 ≤ *RI* < 600; and (IV) high risk: *RI* ≥ 600:

$$RI=\sum Er^{i}$$

#### Monte Carlo simulation

The probability distribution of *$E_{r}^{i}$* and *RI* values was obtained by using Monte Carlo simulation [29]. The measured environmental concentrations of each metal in the sediments were used as a data set comprised of random variables that conform to a certain probability distribution. The commonly used cumulative probability distribution functions, which mainly include Weibull, log-normal, log—logistic, and Burr III methods, were all applied for the fitting of the data set. The most suitable model was selected based on the Kolmogorov-Smirnov test (S1 Table): the closer the *P* value is to 1, the better is the fitting effect. When the Burr III distribution was used, its limit distribution appeared, and the fitting effect was poor. In addition, Burr III distribution was often recommended for the species sensitivity distribution (SSD) model fitting [30], but not for that of environmental monitoring data. Therefore, Burr III distribution is not included in S1 Table. Therefore, the log-logistic distribution was found to be the most suitable; the parameters of this distribution are presented in S2 Table. Monte Carlo simulations were performed for 100,000 times using the MATLAB 2017b software.

#### Hazard quotient

Hazard quotient is the quotient of the environmental exposure concentration (EEC) and predicted no effect concentration (PNEC). While HQ > 1 indicates potential ecological risk, HQ < 1 suggests that the ecological risk is at an acceptable level [26, 31, 32]. In this study, the geometric mean of the heavy metal concentrations detected in the seawater were used as the EEC to calculate the HQ in general. The PNEC value was calculated as HC_5_ (hazardous concentration affecting 5% of species) divided by the safety factor (SF = 5) [33, 34]. The value of HC_5_ was derived from the SSD [26, 31]. The above-mentioned cumulative probability distribution functions were applied to derive SSD. The most suitable model was selected based on the Anderson-Darling test (S3 Table): the closer the *P* value is to 1 and the smaller the Akaike information criterion (AIC), the better is the fitting effect [31, 35, 36]. Overall, the log-logistic distribution was found to be the most suitable, and its parameters are presented in S4 Table.

#### Joint probability curve

The same methods and criteria used for selecting the probability distribution model applied to measured concentrations of heavy metals in the surface sediments were also applied to that of the seawater. The results were similar as well, i.e., the fitting results of the log-logistic distribution model were better than those of the log-normal and Weibull methods (S5 and S6 Tables), which were adopted in the JPC construction. The JPC was generated using the cumulative probability of the toxicity data from the SSD as an independent variable and the reverse cumulative probability of the exposure data (or exceedance probability, EXP) as the dependent variable to describe the probability of a certain proportion of species expected to be adversely affected [17]. The distance between the generated curve and the axes positively indicated the risk level, and the area under the curve showed the overall risk probability (ORP) of the adverse effects:

ORP=∑EXP(x)dx

where *x* is the proportion of adversely affected species, and *EXP*(*x*) is the exceedance probability of the exposure data associated with 100*x*% of the adversely affected species.

## Results and discussion

### Measured concentrations of heavy metals in the surface sediments of western Laizhou Bay

The concentrations (mg/kg) of heavy metals in the surface sediments of western Laizhou Bay were in the range of 9.20–12.70 (average 11.01) for As, 0.11–0.18 (average 0.16) for Cd, 23.60–37.00 (average 30.40) for Cr, 17.60–25.50 (average 20.36) for Cu, 0.009–0.035 (average 0.019) for Hg, 13.40–24.60 (average 17.65) for Pb, and 21.50–43.50 (average 30.21) for Zn (Tables 4 and 5). The concentrations of heavy metals detected in the surface sediments of western Laizhou Bay met Grade I of the Marine Sediment Quality (GB 18668–2002), and were comparable to (for As, Cd, Cr, Cu, and Pb) or lower than (for Hg and Zn) those of other coastal systems around the world. The results of the matched-pair t-test (pair of May-Sep) showed significant differences for all the seven elements. Thus, the two data sets were separately analyzed in the following ecological risk assessment (S7 Table).

**Table 4:** Measured concentrations of heavy metals in the surface sediments of western Laizhou Bay (unit: mg/kg).

Matter	2016.05			2016.09		
	Range	Mean	SD	Range	Mean	SD
As	10.70–12.70	11.49	0.71	9.20–11.90	10.50	0.78
Cd	0.11–0.18	0.14	0.02	0.16–0.18	0.17	0.01
Cr	23.60–29.80	26.51	1.87	31.50–37.00	34.30	1.71
Cu	18.00–25.50	21.75	2.25	17.60–20.50	19.30	0.88
Hg	0.009–0.016	0.011	0.002	0.022–0.035	0.025	0.005
Pb	17.50–24.60	20.53	2.35	13.40–15.80	14.80	0.93
Zn	21.50–35.50	27.42	3.91	34.40–43.50	39.00	2.81

SD: standard deviation.

**Table 5:** Mean concentrations of heavy metals in the surface sediments of western Laizhou Bay compared to those of other coastal systems around the world (unit: mg/kg).

Location	As	Cd	Cr	Cu	Hg	Pb	Zn	Reference
Masan Bay, Korea	ND	ND	67.1	43.4	ND	44	206.3	[37]
Bremen Bay, Germany	ND	ND	131	87	ND	122	206.3	[38]
Izmit Bay, Turkey	ND	6.3	81.7	89.4	ND	94.9	ND	[39]
Egypt Bay, USA	ND	0.44	0.39	14.2	ND	27	77.5	[40]
Liaodong Bay, China	8.3	NA	46.4	19.4	0.04	31.8	71.7	[41]
North Yellow Sea, China	ND	0.09	48.9	14.44	ND	24.1	57.3	[42]
Bohai Bay, China	ND	0.12	68.6	24	ND	25.6	73	[43]
Central Bohai Sea, China	ND	0.14	61.45	24.34	ND	30.69	79.91	[44]
Southwestern Laizhou Bay, China	10.05	ND	ND	ND	0.035	ND	ND	[12]
Laizhou Bay, China	7.1	0.19	32.69	10.99	0.039	13.37	50.63	[14]
Laizhou Bay, China	12.7	0.12	60.0	22.0	ND	21.9	60.4	[15]
Western Laizhou Bay, China	11.01	0.16	30.40	20.36	0.019	17.65	30.21	This study

ND: not detected.

### Ecological risk of heavy metals in the surface sediments of western Laizhou Bay

**Fig 2:**
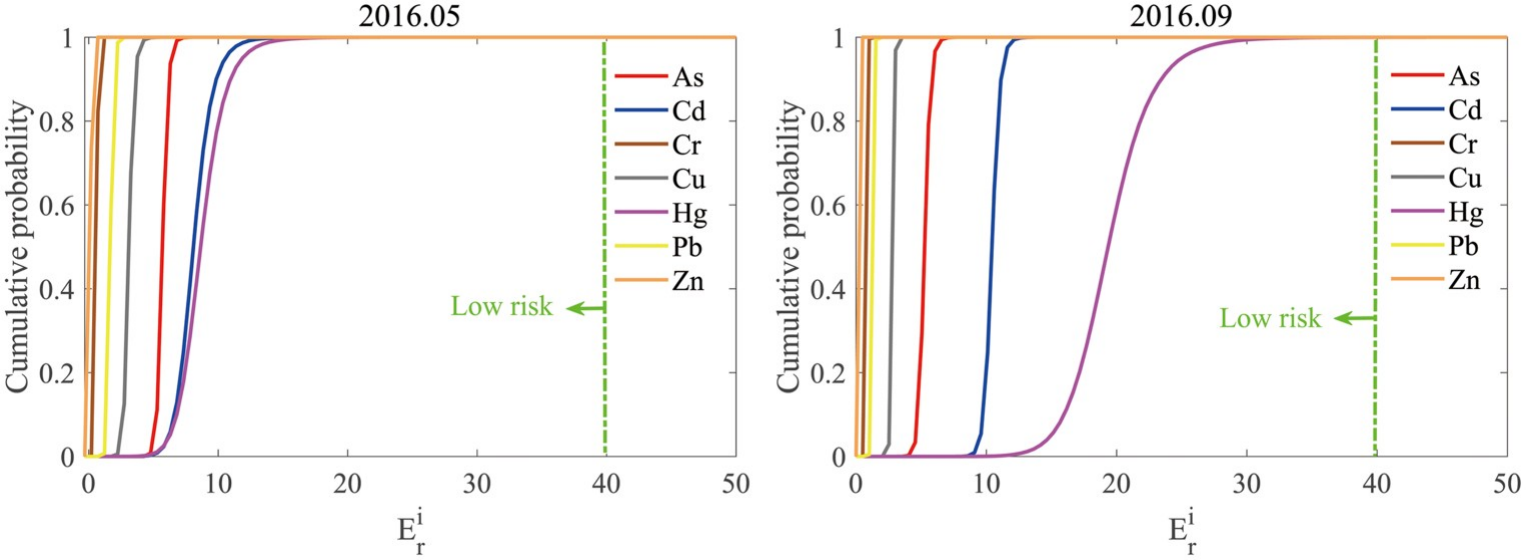
Cumulative probability curves of $E_{r}^{i}$ for each heavy metal in the surface sediments of western Laizhou Bay. $E_{r}^{i}$ is the potential ecological risk factor of substance ‘‘*i*”, and the green dotted line represents $E_{r}^{i}$ = 40. Cumulative probability curves of $E_{r}^{i}$ on the left side of this straight line indicate low risk.

By applying a Monte Carlo simulation, the ecological risks of each heavy metal and the mixture in the surface sediments of western Laizhou Bay were expressed as a probability distribution of $E_{r}^{i}$ and *RI* values instead of single-point estimates. Fig 2 shows the cumulative probability curves of $E_{r}^{i}$ for each heavy metal. Apparently, the $E_{r}^{i}$ curves of Hg, Cd, and As are towards the right compared to those of Cr, Cu, Pb, and Zn; however, all curves are to the left of the straight line $E_{r}^{i}=40$, indicating low risk. The Monte Carlo simulation demonstrated that only Hg in September 2016 showed a probability (0.03%) of moderate risk (Table 6). The sources of Hg in this area were mainly land-based human activities, including factory discharge and combustion of fossil fuels, and river transportation is possibly the main means by which Hg enters Laizhou Bay [12, 14].

**Table 6:** Ecological risk for each heavy metal in the surface sediments of western Laizhou Bay.

Time	Matter	Average estimation		Probability of each grade based on Monte Carlo (%)				
		Average *$E_{r}^{i}$*	Grade from $E_{r}^{i}$	Low	Moderate	Considerable	High	Very high
2016.05	As	5.75	Low	100	0	0	0	0
	Cd	8.22	Low	100	0	0	0	0
	Cr	0.66	Low	100	0	0	0	0
	Cu	3.11	Low	100	0	0	0	0
	Hg	8.88	Low	100	0	0	0	0
	Pb	1.71	Low	100	0	0	0	0
	Zn	0.18	Low	100	0	0	0	0
2016.09	As	5.25	Low	100	0	0	0	0
	Cd	10.44	Low	100	0	0	0	0
	Cr	0.86	Low	100	0	0	0	0
	Cu	2.76	Low	100	0	0	0	0
	Hg	20.24	Low	99.97	0.03	0	0	0
	Pb	1.23	Low	100	0	0	0	0
	Zn	0.26	Low	100	0	0	0	0

$E_{r}^{i}$: the potential ecological risk factor of substance ‘‘i”.

**Fig 3:**
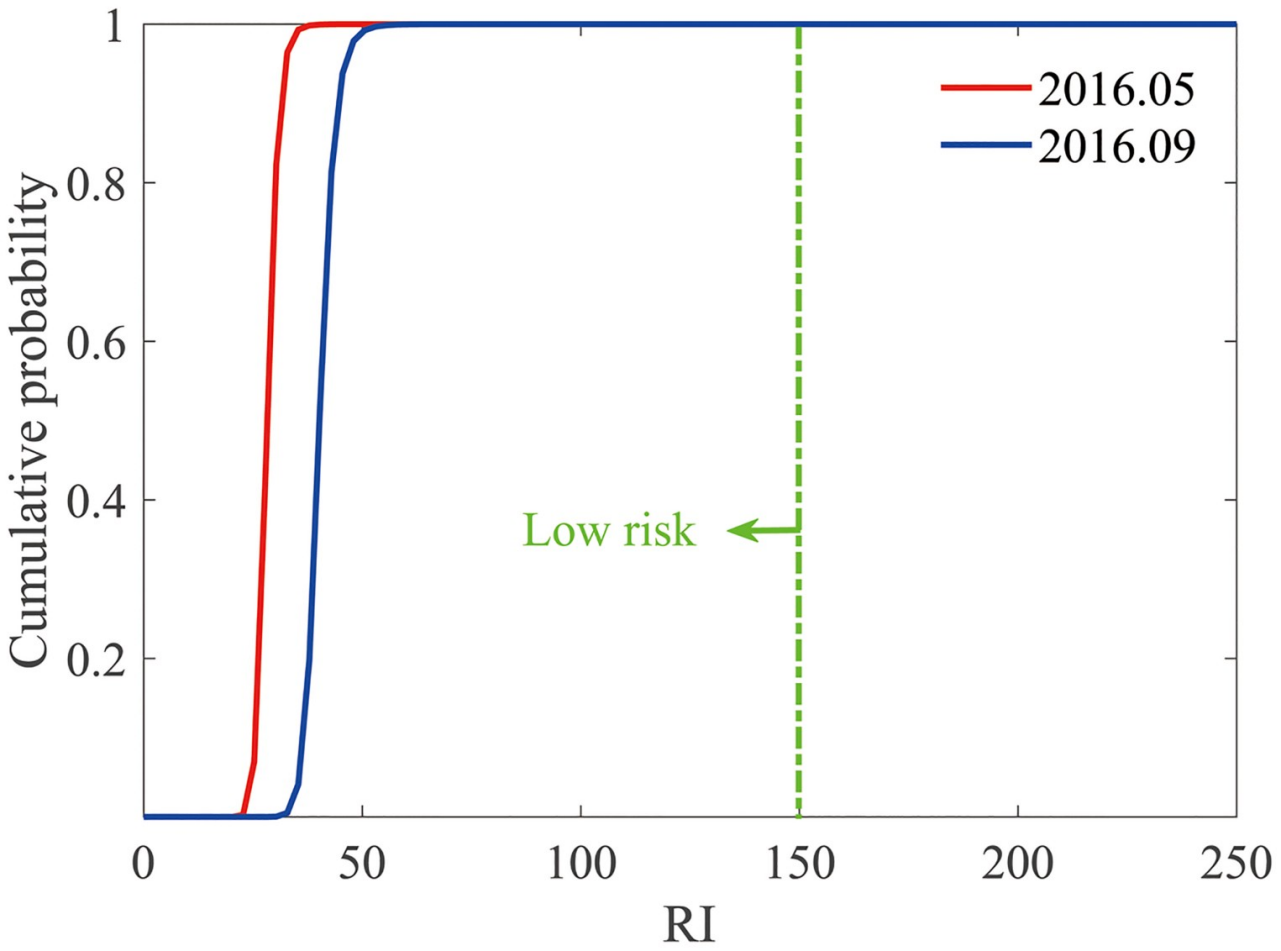
Cumulative probability curves of *RI* of the seven heavy metals in the surface sediments of western Laizhou Bay. ***RI* is the ecological risk for the sediment, and the green dotted line represents *RI*** = **150**. Cumulative probability curves of *RI* on the left side of this straight line indicate low risk.

The *RI* value was 28.50 and 41.04 for May and September 2016, respectively, suggesting low risk for the sediments of western Laizhou Bay. The Monte Carlo simulation also showed that the combined ecological risk caused by these seven metals is 100% low (Fig 3).

Generally, the average, conservative, or maximum values were adopted in typical risk assessment indices, to estimate the average, conservative, or the worst case of ecological risk [45, 46]; by doing this, the risks might be either under- or overestimated. For example, in this study, although the average estimation of $E_{r}^{i}$ for Hg in September 2016 was 20.24, which is only 0.51 times of 40 (the upper limit of the low risk), a probability (0.03%) of moderate risk still existed (Table 6); similarly, although high risk was identified in the Xiangjiang River and Dianchi Lake according to the average *RI* values, the Monte Carlo simulation indicated that the probabilities of considerable risk level reached only as high as 43.3% in the Xiangjiang River and 47.1% in the Dianchi Lake [29]. The combined approach using PERI and Monte Carlo simulation joint approach may therefore avoid either under- or overestimation of the ecological risk and provide a more objective scientific evidence for the environmental management of the polluted aquatic bodies. Furthermore, when the potential ecological risks of heavy metals in soil in a study area in the urban—rural transition zone of the Wuhan City, China was characterized, the spatial distribution of heavy metal was simulated using sequential Gaussian simulation (SGS), which is also a Monte Carlo method, and then the simulated realizations was fed into the Hakanson PERI computation equation to obtain the response maps of the PERI for each metal [47]. Combining SGS or other geostatistical stochastic simulation methods and the Hakanson PERI may be a way for assessing the spatial distribution and uncertainty of the potential ecological risk of heavy metals in sediments.

### Measured concentrations of heavy metals in the surface seawater of western Laizhou Bay

The heavy metal levels in the surface seawater of western Laizhou Bay were 3.01–3.87 μg/L for As, 0.11–0.20 μg/L for Cd, 4.16–5.78 μg/L for Cr, 1.31–2.96 μg/L for Cu, 0.01–0.04 μg/L for Hg, 1.29–2.87 μg/L for Pb, and 30.90–49.80 μg/L for Zn. The average values of the data monitored for the two time periods, i.e., May and September 2016, were 3.50 μg/L for As, 0.16 μg/L for Cd, 5.07 μg/L for Cr, 2.48 μg/L for Cu, 0.03 μg/L for Hg, 1.75 μg/L for Pb, and 40.26 μg/L for Zn (Table 7). Histograms of the sample data for the heavy metals in the surface seawater are shown in S1 and S2 Figs. The concentrations of As, Cd, Cr, Cu, and Hg met Grade I of the Sea Water Quality Standard (GB 3097–1997), while those of Pb and Zn met Grade II. According to the results of the matched-pair t-test (pair of May-Sep) (S8 Table), the concentrations of Cd and Cu in September were significantly higher than those in May (*P* < 0.05). Hence, the ecological risks were separately assessed for the two time points.

**Table 7:** Measured concentrations of heavy metals in the surface seawater of western Laizhou Bay (unit: μg/L).

Matter	2016.05			2016.09		
	Range	Mean	SD	Range	Mean	SD
As	3.01–3.87	3.43	0.26	3.27–3.84	3.57	0.16
Cd	0.11–0.19	0.14	0.02	0.16–0.20	0.18	0.01
Cr	4.16–6.17	5.12	0.63	4.78–5.19	5.01	0.12
Cu	1.31–2.96	2.38	0.38	2.44–2.82	2.58	0.12
Hg	0.01–0.04	0.03	0.01	0.02–0.03	0.02	0.002
Pb	1.29–2.87	1.91	0.49	1.36–1.79	1.58	0.11
Zn	30.90–49.80	40.48	5.35	36.80–43.60	39.85	1.67

SD: standard deviation.

### Ecological risk of heavy metals in the surface seawater of western Laizhou Bay

The ecological risk of heavy metals to the marine ecosystems should include the effect of their deposition in the sediments and resuspension into the surface seawater. However, compared to the various index approaches available for the ecological risk assessment of heavy metals in the sediments, methods for those in the seawater are very limited. Typically, only Single Factor and Nemerow index methods are available. In recent years, new approaches were developed based on the SSD theory [48, 49], and HQ, which is the simplest among them, was adopted in this study. The results showed that HQ was less than or equal to 1 for Cd, Hg, and Pb, indicating that their potential ecological risk was acceptable, whereas HQ was greater than 1 for As, Cr, Cu, and Zn, suggesting unacceptable ecological risk (Table 8).

**Table 8:** Hazard quotient values for heavy metals in the surface seawater of western Laizhou Bay.

Matter	HC_5_ (μg/L)	PNEC (μg/L)	HQ (2016.05)	HQ (2016.09)
As	9.33	1.87	1.83	1.91
Cd	2.57	0.51	0.28	0.35
Cr	1.16	0.23	22.27	21.78
Cu	0.87	0.17	14.00	15.18
Hg	1.08	0.22	0.12	0.11
Pb	9.53	1.91	1.00	0.83
Zn	29.09	5.82	6.96	6.85

HC_5_: hazardous concentration affecting 5% of species; PNEC: predicted no effect concentration; HQ: hazard quotient.

**Fig 4:**
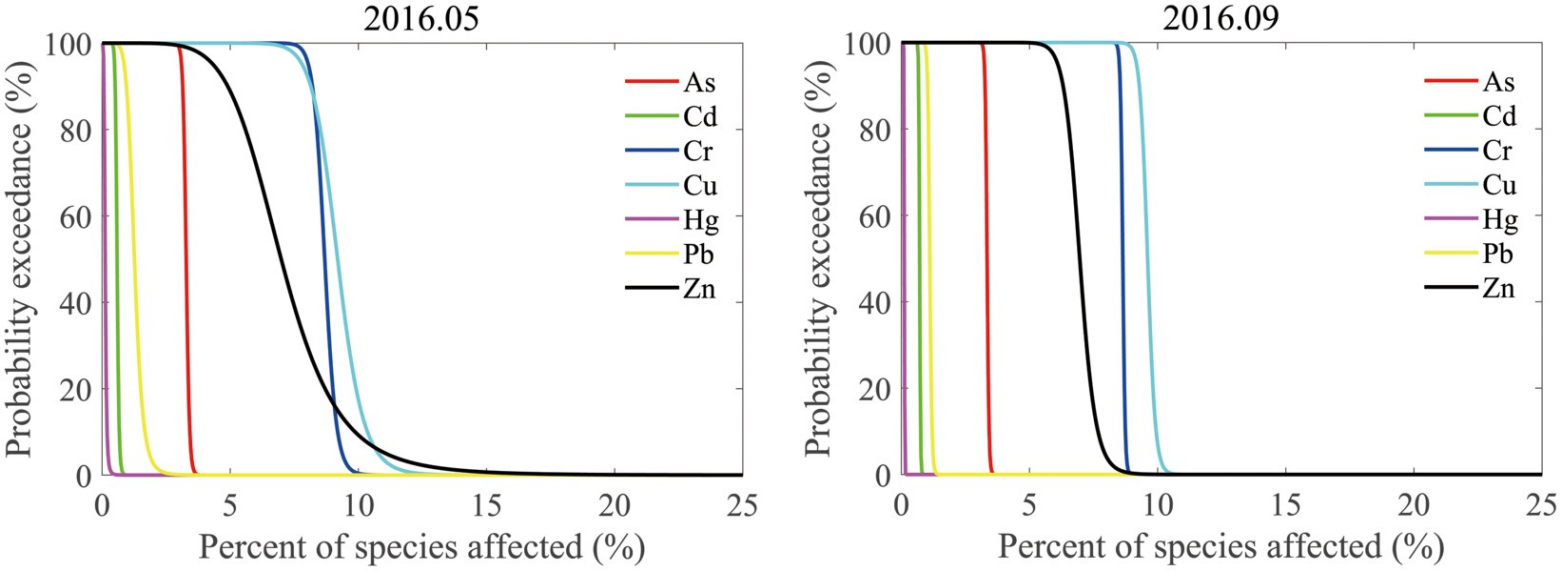
Joint probability curves of heavy metals in the surface seawater of western Laizhou Bay. The distance between the generated curve and the axes positively indicates the risk level, and the area under the curve shows the overall risk probability of the adverse effects.

Although the HQ method has its advantages such as simplicity and low data requirements [25, 50], it is a screening-level method applied to focus on the most dominant pollutants. HQ > 1 does not necessarily demonstrate the real risk of As, Cr, Cu, and Zn. Refinement with higher level methods should follow to alleviate uncertainty to an acceptable degree. Therefore, in this study, the JPC method was employed to refine the aquatic ecological risk assessment. For As, Cr, Cu, and Zn, the distances between their JPC curves and the axes were further than those for the other three elements, indicating their higher risk level (Fig 4). Generally, the ORP of the adverse effects is considered to be acceptable when it is not higher than 0.05 [51]. However, the ORP of Cr, Cu, and Zn ranged from 0.086–0.087, 0.092–0.096, and 0.070–0.073, respectively, suggesting certain ecological risk. This was consistent with the results of HQ. In the case of As, although its HQ was also greater than 1, its ORP was < 0.05, suggesting an overestimation of its ecological risk in the water column by HQ (Table 9). The sources of Cu and Zn were mainly from natural contribution, including coastal erosion, weathering products carried by the surrounding short rivers, and loess matters carried by the Yellow River, while those of Cr were likely from anthropogenic discharges such as metal processing, fuel burning, and domestic sewage, in addition to natural inputs [13–15].

**Table 9:** Overall risk probabilities calculated from joint probability curves for the seven heavy metals in the surface seawater of western Laizhou Bay.

Matter	2016.05	2016.09
As	0.033	0.034
Cd	0.006	0.007
Cr	0.087	0.086
Cu	0.092	0.096
Hg	0.001	0.001
Pb	0.013	0.011
Zn	0.073	0.070

According to the Marine Functional Zoning of Shandong Province (2011–2020), Grade I (for site 1, 2, 3, and 6 in the protection zone) or Grade II (for most sites except for those in the protection zone) of the Sea Water Quality Standard (GB 3097–1997) should be adopted in the study area. Chemical monitoring showed that concentrations of Cr and Cu met Grade I, while the Zn levels met Grade II. However, according to the results of HQ and JPC, Cr, Cu, and Zn posed potential ecological risk to the aquatic environment of western Laizhou Bay, while Cr and Cu were considered as the main aquatic pollutants. The HC_5_ value of Zn was higher than Grade I and lower than Grade II, while that of Cr and Cu was lower than Grade I. The criterion continuous concentrations (CCCs) suggested by US EPA [52] were 50 μg/L for Cr, 3.1 μg/L for Cu, and 81 μg/L for Zn, respectively, and the HC_5_ values of Cr, Cu, and Zn were 0.02, 0.28, and 0.36 times each CCC, respectively, showing certain differences. Mu et al. [53] reported a HC_5_ value of 1.8 μg/L for Cd using chronic toxicity data from local marine organisms in Bohai Bay, which was slightly less than the value of 2.57 μg/L obtained in this study. Thus, the thresholds for environmental protection derived from the chronic toxicity data in this study are observed to be relatively low, and the risk assessment using these values can better characterize the ecological risk posed by the heavy metals in western Laizhou Bay.

On the one hand, uncertainty is inevitable in ecological risk assessment even when high tier approaches are conducted. This could be due to the following reasons. First, physical and chemical parameters (such as hardness, pH, and suspended solid) may affect the distribution and bioavailability of heavy metals in the water environment, and ultimately affect their toxicities to aquatic organisms [54]. Second, the toxicity data used for the construction of SSDs are from marine organisms all over the world, instead of a pool of the local species in the study area. For example, 38 d LOEC of H_2_CrO_4_ to the sensitive species *Palaemon elegans* (0.003 μg/L) and 14 d LOEC of H_2_CrO_4_ to the sensitive species *Palaemonetes varians* (0.001 μg/L) were adopted when the HC_5_ of Cr was calculated [55]. Thus, a high HQ was derived for Cr, and the fitting effect of the SSD curve (especially the tail) was also influenced. On the other hand, the spatial distribution of pollution risks was not mapped in this study. Since sampling in marine environments is difficult and expansive, 20 stations were set in this study, which were comparable to those in other studies focusing on heavy metals or other pollutants in western or southwestern Laizhou Bay [12, 13, 16, 56]. However, the sample data is limited when the variogram estimation is conducted. If more sample data is available especially when a wider sea area is studied, the SGS can be used to conduct a stochastic spatial simulation and map the pollution risks of heavy metals [25, 47, 57, 58].

## Conclusion

The presence of the heavy metals namely As, Cd, Cr, Cu, Hg, Pb, and Zn was detected in the surface sediments and seawater of western Laizhou Bay during the spring and autumn of 2016; and their concentrations were found to be comparable to or lower than those in other coastal areas around the world. The typical potential ecological risk index and Monte Carlo simulation suggested low risk for the sediments of western Laizhou Bay, with the exception of Hg during September 2016, which showed the probability of a moderate risk. The HQ and JPC indicated certain ecological risk for Cr, Cu, and Zn, and acceptable risk for Cd, Hg, Pb, and As in the surface seawater. The ecological risk of heavy metals in western Laizhou Bay was better characterized in this study by applying the probabilistic approaches.

## Supporting information

S1 Fig Histograms of the sample data for the heavy metals in the surface seawater of western Laizhou Bay on May 2016. (TIF)

S2 Fig Histograms of the sample data for the heavy metals in the surface seawater of western Laizhou Bay on September 2016. (TIF)

S1 Table Criteria for model selection for measured concentrations of heavy metals in the surface sediments of western Laizhou Bay based on Kolmogorov-Smirnov test. (DOCX)

S2 Table Parameters of log-logistic distribution model for measured concentrations of heavy metals in the surface sediments of western Laizhou Bay. (DOCX)

S3 Table Criteria for model selection for species sensitivity distribution models and HC_5_ for measured concentrations of heavy metals in the surface seawater of western Laizhou Bay. (DOCX)

S4 Table Parameters of SSD models (log-logistic distribution) for measured concentrations of heavy metals in the surface seawater of western Laizhou Bay. (DOCX)

S5 Table Criteria for model selection for measured concentrations of heavy metals in the surface seawater of western Laizhou Bay based on Kolmogorov-Smirnov test. (DOCX)

S6 Table Parameters of log-logistic distribution model for measured concentrations of heavy metals in the surface seawater of western Laizhou Bay. (DOCX)

S7 Table Matched data t-test (pair of May-September) for seasonal differences in the concentrations of heavy metals in the surface sediments of western Laizhou Bay. (DOCX)

S8 Table Matched data t-test (pair of May-September) for seasonal differences in the concentrations of heavy metals in the surface seawater of western Laizhou Bay. (DOCX)

S1 Dataset (XLSX)
