# Supplementary material for: Probabilistic ecological risk assessment of heavy metals in western Laizhou Bay, Shandong Province, China
Source: PLoS One. 2019 Mar 14;14(3):e0213011. doi: 10.1371/journal.pone.0213011 (PMC6417698; doi:10.1371/journal.pone.0213011)
Supplement: S1 Table — (DOCX) [file pone.0213011.s003.docx]

**S3 Table Criteria for model selection for measured concentrations of heavy metals in the surface sediments of western Laizhou Bay based on Kolmogorov-Smirnov test.**

| **Matter** | **Distribution** | **2016.05** | | **2016.09** | |
| --- | --- | --- | --- | --- | --- |
|  |  | ***P* value** | **K-S stat** | ***P* value** | **K-S stat** |
| As | Log-normal | 0.75 | 0.20 | 0.98 | 0.16 |
|  | Log-logistic | 0.71 | 0.21 | 0.99 | 0.15 |
|  | Weibull | 0.59 | 0.23 | 0.84 | 0.22 |
| Cd | Log-normal | 0.77 | 0.20 | 0.73 | 0.24 |
|  | Log-logistic | 0.87 | 0.17 | 0.82 | 0.22 |
|  | Weibull | 0.44 | 0.26 | 0.94 | 0.19 |
| Cr | Log-normal | 0.98 | 0.13 | 0.88 | 0.20 |
|  | Log-logistic | 0.99 | 0.13 | 0.92 | 0.19 |
|  | Weibull | 0.91 | 0.16 | 0.63 | 0.26 |
| Cu | Log-normal | 0.86 | 0.18 | 0.81 | 0.22 |
|  | Log-logistic | 0.88 | 0.17 | 0.96 | 0.18 |
|  | Weibull | 0.92 | 0.16 | 0.99 | 0.15 |
| Hg | Log-normal | 0.97 | 0.14 | 0.21 | 0.38 |
|  | Log-logistic | 0.99 | 0.13 | 0.39 | 0.32 |
|  | Weibull | 0.90 | 0.17 | 0.25 | 0.36 |
| Pb | Log-normal | 0.47 | 0.25 | 0.74 | 0.24 |
|  | Log-logistic | 0.69 | 0.21 | 0.85 | 0.21 |
|  | Weibull | 0.30 | 0.29 | 0.86 | 0.21 |
| Zn | Log-normal | 0.88 | 0.17 | 0.99 | 0.14 |
|  | Log-logistic | 0.95 | 0.15 | 0.99 | 0.15 |
|  | Weibull | 0.71 | 0.21 | 0.98 | 0.16 |

K-S stat: statistics of Kolmogorov-Smirnov test.
