# Supplementary material for: Probabilistic ecological risk assessment of heavy metals in western Laizhou Bay, Shandong Province, China
Source: PLoS One. 2019 Mar 14;14(3):e0213011. doi: 10.1371/journal.pone.0213011 (PMC6417698; doi:10.1371/journal.pone.0213011)
Supplement: S2 Table — (DOCX) [file pone.0213011.s004.docx]

**S4 Table Parameters of log-logistic distribution model for measured concentrations of heavy metals in the surface sediments of western Laizhou Bay.**

| **Matter** | **2016.05** | | **2016.09** | |
| --- | --- | --- | --- | --- |
|  | ***μ* (95% CI)** | ***σ* (95% CI)** | ***μ* (95% CI)** | ***σ* (95% CI)** |
| As | 2.43 (2.39–2.47) | 0.04 (0.02–0.06) | 2.35 (2.30–2.41) | 0.04 (0.02–0.08) |
| Cd | –2.01 (–2.10–(–1.91)) | 0.09 (0.05–0.15) | –1.75 (–1.99–(–1.71)) | 0.03 (0.02–0.05) |
| Cr | 3.27 (3.23–3.32) | 0.04 (0.03–0.07) | 3.53 (3.50–3.57) | 0.03 (0.02–0.05) |
| Cu | 3.08 (3.01–3.15) | 0.06 (0.04–0.10) | 2.96 (2.93–3.00) | 0.03 (0.01–0.05) |
| Hg | –4.53 (–4.65–(–4.41) | 0.11 (0.07–0.18) | –3.72 (–3.83–(–3.61)) | 0.09 (0.05–0.17) |
| Pb | 3.00 (2.93–3.08) | 0.07 (0.04–0.11) | 2.70 (2.65–2.75) | 0.04 (0.02–0.07) |
| Zn | 3.31 (3.22–3.39) | 0.08 (0.05–0.14) | 3.66 (3.61–3.72) | 0.04 (0.02–0.08) |

*μ*: mean of logarithmic values; *σ*: scale parameter of logarithmic values; CI: confidence interval.
