# Supplementary material for: Probabilistic ecological risk assessment of heavy metals in western Laizhou Bay, Shandong Province, China
Source: PLoS One. 2019 Mar 14;14(3):e0213011. doi: 10.1371/journal.pone.0213011 (PMC6417698; doi:10.1371/journal.pone.0213011)
Supplement: S3 Table — (DOCX) [file pone.0213011.s005.docx]

**S5 Table Criteria for model selection for species sensitivity distribution models and HC_5_ for measured concentrations of heavy metals in the surface seawater of western Laizhou Bay.**

| **Matter** | **Distribution** | ***P* value (A-D stat)** | **AIC** | **HC_5_ (95% CI)** |
| --- | --- | --- | --- | --- |
| As | Log-normal | 0.76 (0.49) | 535.6 | 13.22 (1.98–176.39) |
|  | Log-logistic | 0.76 (0.49) | 537.6 | 9.33 (0.87–218.16) |
|  | Weibull | 0.83 (0.41) | 534.6 | 3.54 (0.19–109.94) |
|  | Burr III | 0.78 (0.47) | 539.3 | 18.34 (4.58–157.89) |
| Cd | Log-normal | 0.84 (0.40) | 736.8 | 2.55 (0.87–7.77) |
|  | Log-logistic | 0.90 (0.34) | 739.7 | 2.57 (0.91–7.22) |
|  | Weibull | 0.23 (1.30) | 749.9 | 0.32 (0.09–1.87) |
|  | Burr III | 0.91 (0.33) | 743.6 | 2.84 (0.69–10.57) |
| Cr | Log-normal | 0.35 (1.01) | 779.7 | 0.66 (0.03–17.76) |
|  | Log-logistic | 0.50 (0.77) | 777.1 | 1.16 (0.07–13.21) |
|  | Weibull | 0.34 (1.04) | 774.9 | 0.16 (0.01–2.85) |
|  | Burr III | 0.45 (0.84) | 778.2 | 0.45 (0.01–14.33) |
| Cu | Log-normal | 0.01 (3.67) | 1777.1 | 0.80 (0.42–1.66) |
|  | Log-logistic | 0.09 (2.01) | 1765.8 | 0.87 (0.40–1.93) |
|  | Weibull | <0.01 (8.96) | 1830.5 | 0.01 (0.005–0.045) |
|  | Burr III | 0.21 (1.36) | 1760.0 | 1.86 (0.93–3.88) |
| Hg | Log-normal | 0.93 (0.31) | 206.2 | 1.04 (0.37–3.93) |
|  | Log-logistic | 0.98 (0.24) | 205.5 | 1.08 (0.31–4.07) |
|  | Weibull | 0.44 (0.86) | 212.3 | 0.13 (0.03–2.44) |
|  | Burr III | 0.98 (0.24) | 207.4 | 1.19 (0.00–5.63) |
| Pb | Log-normal | 0.53 (0.73) | 467.9 | 7.21 (1.65–35.76) |
|  | Log-logistic | 0.88 (0.36) | 464.2 | 9.53 (2.18–34.58) |
|  | Weibull | 0.12 (1.78) | 477.3 | 0.46 (0.09–14.85) |
|  | Burr III | 0.89 (0.36) | 466.2 | 10.32 (1.39–52.81) |
| Zn | Log-normal | 0.96 (0.26) | 822.4 | 30.36 (16.59–60.88) |
|  | Log-logistic | 0.98 (0.23) | 822.8 | 29.09 (15.43–57.48) |
|  | Weibull | 0.37 (0.98) | 830.0 | 9.02 (4.20–23.70) |
|  | Burr III | 0.98 (0.22) | 824.7 | 31.51 (13.40–77.13) |

HC_5_: hazardous concentration affecting 5% of species; A-D stat: statistics of Anderson-Darling, AIC: Akaike information criterion; CI: confidence interval.
