# Supplementary material for: Probabilistic ecological risk assessment of heavy metals in western Laizhou Bay, Shandong Province, China
Source: PLoS One. 2019 Mar 14;14(3):e0213011. doi: 10.1371/journal.pone.0213011 (PMC6417698; doi:10.1371/journal.pone.0213011)
Supplement: S4 Table — (DOCX) [file pone.0213011.s006.docx]

**S6 Table Parameters of SSD models (log**-**logistic distribution) for measured concentrations of heavy metals in the surface seawater of western Laizhou Bay.**

| **Matter** | ***μ*** **(95% CI)** | ***σ* (95% CI)** |
| --- | --- | --- |
| As | 8.98 (7.31–10.66) | 2.29 (1.65–3.19) |
| Cd | 4.86 (4.23–5.50) | 1.33 (1.06–1.68) |
| Cr | 7.49 (6.12–8.85) | 2.49 (1.91–3.25) |
| Cu | 3.08 (3.01–3.15) | 0.06 (0.04–0.10) |
| Hg | 3.00 (2.25–3.75) | 0.99 (0.69–1.44) |
| Pb | 5.68 (4.97–6.40) | 1.16 (0.85–1.59) |
| Zn | 5.99(5.57–6.41) | 0.89(0.71–1.11) |

*μ*: mean of logarithmic values; *σ*: scale parameter of logarithmic values; CI: confidence interval.

Probability density function (PDF) of log-logistic distribution is $f(x\left| \mu,\sigma\right.)=\frac{1}{\sigma x}\frac{e^{\frac{\log(x)-\mu}{\sigma}}}{\left( 1+e^{\frac{\log(x)-\mu}{\sigma}} \right)^{2}}$
