# Supplementary material for: Probabilistic ecological risk assessment of heavy metals in western Laizhou Bay, Shandong Province, China
Source: PLoS One. 2019 Mar 14;14(3):e0213011. doi: 10.1371/journal.pone.0213011 (PMC6417698; doi:10.1371/journal.pone.0213011)
Supplement: S5 Table — (DOCX) [file pone.0213011.s007.docx]

**S7 Table Criteria for model selection for measured concentrations of heavy metals in the surface seawater of western Laizhou Bay based on Kolmogorov-Smirnov test.**

| **Matter** | **Distribution** | **2016.05** | | **2016.09** | |
| --- | --- | --- | --- | --- | --- |
|  |  | ***P* value** | **K-S stat** | ***P* value** | **K-S stat** |
| As | Log-normal | 0.71 | 0.15 | 0.98 | 0.10 |
|  | Log-logistic | 0.77 | 0.14 | 0.98 | 0.10 |
|  | Weibull | 0.59 | 0.17 | 0.98 | 0.10 |
| Cd | Log-normal | 0.67 | 0.15 | 0.80 | 0.14 |
|  | Log-logistic | 0.67 | 0.15 | 0.98 | 0.10 |
|  | Weibull | 0.58 | 0.17 | 0.86 | 0.13 |
| Cr | Log-normal | 0.98 | 0.10 | 0.80 | 0.14 |
|  | Log-logistic | 0.97 | 0.10 | 0.81 | 0.14 |
|  | Weibull | 0.86 | 0.13 | 0.48 | 0.18 |
| Cu | Log-normal | 0.86 | 0.13 | 0.43 | 0.19 |
|  | Log-logistic | 0.95 | 0.11 | 0.59 | 0.17 |
|  | Weibull | 0.91 | 0.12 | 0.33 | 0.20 |
| Hg | Log-normal | 0.22 | 0.23 | 0.85 | 0.13 |
|  | Log-logistic | 0.82 | 0.18 | 0.83 | 0.13 |
|  | Weibull | 0.48 | 0.13 | 0.66 | 0.16 |
| Pb | Log-normal | 0.86 | 0.13 | 0.99 | 0.08 |
|  | Log-logistic | 0.88 | 0.12 | 0.99 | 0.08 |
|  | Weibull | 0.87 | 0.13 | 0.77 | 0.14 |
| Zn | Log-normal | 0.83 | 0.13 | 0.57 | 0.17 |
|  | Log-logistic | 0.89 | 0.12 | 0.62 | 0.16 |
|  | Weibull | 0.45 | 0.18 | 0.51 | 0.18 |

K-S stat: statistics of Kolmogorov-Smirnov test.
