# Supplementary material for: Probabilistic ecological risk assessment of heavy metals in western Laizhou Bay, Shandong Province, China
Source: PLoS One. 2019 Mar 14;14(3):e0213011. doi: 10.1371/journal.pone.0213011 (PMC6417698; doi:10.1371/journal.pone.0213011)
Supplement: S6 Table — (DOCX) [file pone.0213011.s008.docx]

**S8 Table Parameters of log-logistic distribution model for measured concentrations of heavy metals in the surface seawater of western Laizhou Bay.**

| **Matter** | **2016.05** | | **2016.09** | |
| --- | --- | --- | --- | --- |
|  | ***μ* (95% CI)** | ***σ* (95% CI)** | ***μ* (95% CI)** | ***σ* (95% CI)** |
| As | 1.23 (1.19–1.26) | 0.05 (0.03–0.07) | 1.27 (1.25–1.29) | 0.03 (0.02–0.04) |
| Cd | –1.98 (–2.04–(–1.91)) | 0.08 (0.06–0.12) | –1.71 (–1.74–(–1.69)) | 0.03 (0.02–0.05) |
| Cr | 1.63 (1.57–1.68) | 0.07 (0.05–0.10) | 1.61 (1.60–1.62) | 0.01 (0.01–0.02) |
| Cu | 0.87 (0.80–0.94) | 0.10 (0.06–0.13) | 0.94 (0.92–0.96) | 0.03 (0.02–0.04) |
| Hg | –3.67 (–3.83–(–3.51)) | 0.22 (0.15–0.32) | –3.70 (–3.75–(–3.66)) | 0.06 (0.04–0.08) |
| Pb | 0.61 (0.49–0.73) | 0.15 (0.11–0.22) | 0.45 (0.42–0.49) | 0.04 (0.03–0.06) |
| Zn | 3.69 (3.63–3.75) | 0.08 (0.05–0.11) | 3.68 (3.66–3.70) | 0.02 (0.02–0.04) |

*μ*: mean of logarithmic values; *σ*: scale parameter of logarithmic values; CI: confidence interval.
