# Supplementary material for: Probabilistic ecological risk assessment of heavy metals in western Laizhou Bay, Shandong Province, China
Source: PLoS One. 2019 Mar 14;14(3):e0213011. doi: 10.1371/journal.pone.0213011 (PMC6417698; doi:10.1371/journal.pone.0213011)
Supplement: S7 Table — (DOCX) [file pone.0213011.s009.docx]

**S9 Table Matched data t-test (pair of May-September) for seasonal differences in the concentrations of heavy metals in the surface sediments of western Laizhou Bay.**

| **Matter** | **Mean** | ***t*** | ***P*** |
| --- | --- | --- | --- |
| As | 1.157* | 4.057 | 0.007 |
| Cd | –0.027* | –3.931 | 0.008 |
| Cr | 1.654E1* | 23.680 | 0.000 |
| Cu | 3.629* | 3.793 | 0.009 |
| Hg | –0.014* | –7.429 | 0.000 |
| Pb | 6.686* | 7.004 | 0.000 |
| Zn | –1.032E1* | –6.509 | 0.001 |

*Asterisk indicates significant difference (*P* < 0.05).
