# Supplementary material for: Probabilistic ecological risk assessment of heavy metals in western Laizhou Bay, Shandong Province, China
Source: PLoS One. 2019 Mar 14;14(3):e0213011. doi: 10.1371/journal.pone.0213011 (PMC6417698; doi:10.1371/journal.pone.0213011)
Supplement: S8 Table — (DOCX) [file pone.0213011.s010.docx]

**S10 Table Matched data t-test (pair of May-September) for seasonal differences in the concentrations of heavy metals in the surface seawater of western Laizhou Bay.**

| **Matter** | **Mean** | ***t*** | ***P*** |
| --- | --- | --- | --- |
| As | –0.312 | –1.667 | 0.112 |
| Cd | –0.454* | –5.037 | 0.000 |
| Cr | –0.137 | –0.468 | 0.645 |
| Cu | –0.203* | –2.236 | 0.038 |
| Hg | –0.001 | –0.230 | 0.821 |
| Pb | 0.265 | 1.750 | 0.096 |
| Zn | –1.200 | –0.496 | 0.626 |

*Asterisk indicates significant difference (*P* < 0.05).
